# Green Synthesis of Lead Sulphide Nanoparticles for High-Efficiency Perovskite Solar Cell Applications

**DOI:** 10.3390/nano12111933

**Published:** 2022-06-05

**Authors:** Mohammad Aminul Islam, Dilip Kumar Sarkar, Md. Shahinuzzaman, Yasmin Abdul Wahab, Mayeen Uddin Khandaker, Nissren Tamam, Abdelmoneim Sulieman, Nowshad Amin, Md. Akhtaruzzaman

**Affiliations:** 1 Department of Electrical Engineering, Faculty of Engineering, University of Malaya, Jalan Universiti, Kuala Lumpur 50603, Malaysia; 2 Solar Energy Research Institute (SERI), Universiti Kebangsaan Malaysia, Bangi 43600, Malaysia; dilipks551@gmail.com (D.K.S.); shahinchmiu@gmail.com (M.S.); akhtar@ukm.edu.my (M.A.); 3 School of Computer Science and Informational Technology, Central University of Science and Technology, Dhaka 1216, Bangladesh; 4 Nanotechnology & Catalysis Research Centre, University of Malaya, Kuala Lumpur 50603, Malaysia; yasminaw@um.edu.my; 5 Centre for Applied Physics and Radiation Technologies, School of Engineering and Technology, Sunway University, Bandar Sunway 47500, Malaysia; mayeenk@sunway.edu.my; 6 Department of Physics, College of Science, Princess Nourah Bint Abdulrahman University, Riyadh 11671, Saudi Arabia; nmtamam@pnu.edu.sa; 7 Department of Radiology and Medical Imaging, Prince Sattam Bin Abdul Aziz University, Alkharj 11942, Saudi Arabia; a.sulieman@psau.edu.sa; 8 College of Engineering, Universiti Tenaga Nasional (@The National Energy University), Jalan IKRAM-UNITEN, Kajang 43000, Malaysia; nowshad@uniten.edu.my; 9 Department of General Educational Development, Faculty of Science and Information Technology, Daffodil International University, DIU Rd, Dhaka 1341, Bangladesh

**Keywords:** PbS nanoparticle, green synthesis, TEM, PbS thin film, perovskite solar cells

## Abstract

In this study, lead sulfide (PbS) nanoparticles were synthesized by the chemical precipitation method using Aloe Vera extract with PbCl_2_ and Thiourea (H_2_N-CS-NH_2_). The synthesized nanoparticles have been investigated using x-ray diffraction (XRD), UV-Vis, energy-dispersive x-ray spectroscopy (EDX), scanning electron microscopy (SEM), and transmission electron microscopy (TEM). XRD and TEM results confirm that the films are in the cubic phase. The crystallite size, lattice constant, micro-strain, dislocation density, optical bandgap, etc. have been determined using XRD and UV-Vis for investigating the quality of prepared nanoparticles. The possible application of these synthesized nanoparticles in the solar cells was investigated by fabricating the thin films on an FTO-coated and bare glass substrate. The properties of nanoparticles were found to be nearly retained in the film state as well. The experimentally found properties of thin films have been implemented for perovskite solar cell simulation and current-voltage and capacitance-voltage characteristics have been investigated. The simulation results showed that PbS nanoparticles could be a potential hole transport layer for high-efficiency perovskite solar cell applications.

## 1. Introduction

The power conversion efficiency (PCE) of organo-metal-halide perovskite solar cells (PSCs) has already been improved from 3.1% to 25.2% within just 10.0 years, which already exceeds the efficiency of the highest commercialized thin-film solar cells, such as the most dominant CdTe solar cells [1,2]. Due to exceptional optoelectrical features, such as strong light absorption, ease of manufacturing, ambipolar carrier transport character, high mobility, and long diffusion length, perovskite materials in particular offer tremendous promise to work as efficient photovoltaic absorber materials [3,4]. However, operational stability issues such as humidity, temperature, and irradiation stability continue to be important roadblocks in the practical deployment of PSCs [5]. Particularly, the organic part in Perovskite material is prone to decay when exposed to a moist atmosphere [6]. It is believed that the charge transport layer could play a key function in shielding the perovskite layer from moisture [7]. Spiro- OMeTAD is a commonly used hole transport layer (HTL) in high-efficiency PSCs, which degrade itself from the invasion of moisture [8]. Moreover, the presence of a pin-hole in spiro-OMeTAD influences the amount of recombination loss at the perovskite-HTL interface [9]. Numerous alternative materials for HTLs, such as inorganic p-type semiconductors, have been proposed, developed, and employed in PSCs [10,11,12,13,14,15,16,17]. Particularly, those new HTLs were typically fabricated focusing on hydrophobic nature supposed to avoid water vapor transport through and reaction with perovskite materials. However, PSCs based on those HTLs have a lower performance conversion efficiency (PCE) than those based on organic HTLs (such as spiro-OMeTAD), which will certainly obstruct PSC commercialization in the future. Importantly, NiO*_x_* is one of the most thoroughly explored HTLs among the inorganic HTLs due to its easy deposition procedure with high transmittance, larger bandgap, and deep valence band. So far, the highest 21.66% has been reported for NiO*_x_* based PSC [18]. It should be noted that NiOx-based HTLs have some drawbacks, such as limited hole conductivity and poor electrical and/or physical contact with the perovskite [19].

In addition, some of the alternative inorganic HTLs allow metal ion migration through them into the perovskite, which has a significant impact on the device’s performance and stability [20]. An alternative approach, such as inserting a buffer layer in-between HTL and metal electrode, or in between the HTL and perovskite layer, has already been implemented to diminish the above adverse effects. A PSC with an ultrathin compact Al_2_O_3_ buffer layer on top of the HTL fabricated via atomic layer deposition (ALD) technique was reported to retain 90% of its initial PCE after 24 days of air storage [6]. Besides, PSCs with a MoS_2_ [21], MoO*_x_* [22], CuS*_x_* [23], FeS_2_ [6], and Cr [24] buffer layer have also been tested, and significant improvement in the ambient condition has been observed, however, their PCEs are found comparatively lower than those without these buffer layers. Alternatively, Li et al. [25] fabricated a PSC with a PCE of about 8% based on a hole transport layer of Lead sulfide (PbS) colloidal quantum dots (CQDs), demonstrating the potential use of PbS as an efficient inorganic HTL.

Recently, Zheng et al. [26] fabricated a PbS-based PSC with a PCE of 19.58% which retained nearly 100% of its initial PCE after 1000 h of storage in ambient air. Particularly, PbS is a traditional direct bandgap semiconductor with a large excitation Bohr radius (~18 nm), for which its bandgap could be tuned over a wide range by controlling and/or modifying particle size. [27]. Instead, PbS could serve as a light harvester, a newly certified record conversion efficiency of 11.28% has been achieved in CQDs photovoltaics using the PbS CQDs [28]. Besides, PbS nanoparticles could promote the perovskite grains’ growth, resulting in a substantial improvement in the surface morphology and crystallinity of perovskite films [29]. Also, it was reported that the spiro- OMeTAD/PbS bilayer confirmed superior hole mobility which accelerates the carrier extraction towards the HTL and ensures higher PCE [26]. Most importantly, the PbS could provide an efficient permeation barrier against moisture and increase perovskite moisture stability as it is a hydrophobic material, and PbS can hinder the metal migration into the perovskite layer and increase the cell thermal stability [26]. The only issue is that the fabrication procedure needs to be improved since the PbS nanoparticles (NP) solution’s solvent might easily harm the perovskite during spin-coating, as a result, PSC with PbS nanoparticle show limited performance and durability under a normal air environment. Finding acceptable approaches is thus a critical scope that may be accomplished through the use of appropriate solute and/or solution concentration.

Herein, we are first reporting the synthesized PbS nanoparticles using plant extract that could be an alternative favorable material for using as a standalone HTL and/or as a buffer layer between the HTL and the metal electrode in perovskite solar cells. Our focus is on developing PbS nanoparticles using a simple, green, and economic method that is particularly highly desirable. AV is a well-known plant that has about 75 active components that are classified as phytochemicals [30]. Polysaccharides, flavonoids, carbohydrates, coumarins, tannins, chromones, anthraquinones, organic compounds, pyrones, phytosterols, anthrones, sterols, vitamins, proteins, and mineral components are the most commonly discovered AV-phytochemicals [31]. Their organizational structure and functional groups vary. In the synthesis process, these phytochemicals serve as complexing agents, capping agents, and surfactants. It should be noted that the phytochemicals included in the extract are determined by the AV plants (locations, age, body parts, etc.) and the solvents (water, ethanol, DMSO, etc.) employed to prepare the extract [32]. Water-soluble organic components (phytol, sterols, saccharides, alkaloids, etc.) and minerals present in the AV plant make up the majority of the AV water extract. All extract components work together to tune product quality, such as particle size, crystallite forms, morphology, and so on [33,34]. PbCl_2_ and thiourea were used as sources of Pb^2+^ and S^2−^, respectively, in this investigation, with AV-water extract serving as a complexing agent and product-developing phytochemicals. PbCl_2_ is only marginally soluble in water, but at roughly 75 °C, its solubility increases somewhat, and thiourea decomposes to create S^2−^ [35,36]. In that case, AV-extract mediates the formation of PbCl_2_ nanoparticles without the usage of any other chemical (surfactant, dispersion) such as cetyltrimethylammonium bromide (CTAB) [37,38] or ethylene diamine tetraacetic acid (EDTA), which are commonly utilized in traditional PbCl_2_ manufacturing procedures.

We investigate in detail the synthesized PbS nanoparticles using X-ray diffraction (XRD), UV-Vis, Field effect scanning electron microscopy (FESEM), Energy-dispersive X-ray spectroscopy (EDX), and transmission electron microscopy (TEM). A significant impact in structural and optical properties has been observed for two different calcination temperatures. We also fabricated the thin films using these nanoparticles and investigate the viability through XRD, UV-Vis, and Hall Effect measurement as well as the performance of PSC using device simulation.

## 2. Methodology

Fresh AV was collected from Taman Tenaga farmland and washed with DI water then cut into slices. 425 mL DI water and 75 g slices (85 mL DI water for each 15 g of AV slice) were taken into a 1-L beaker, then the mixture was heated around 60 °C with gently stirring for 1 h. Finally, the plant (P)-extract (light grey filtrate) was collected by filtration with Whatman filter paper with a pore size of 11 μm. These various organic substances containing water-AV extract (P-extract) were used for the synthesis of PbS nanomaterial in our modified method.

**Figure 1:**
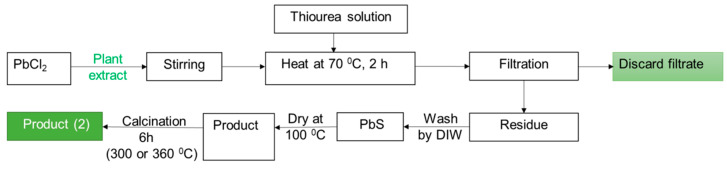
Flow chart for the synthesis of PbS nanomaterial using the Aloe Vera plant extract.

Later on, 2.5 mM equivalent to 0.695 g of PbCl_2_ was taken into a 250 mL volumetric flask and added P-extract up to the mark. The mixture was transferred into a 500 mL beaker and placed on a hot plate to heat at nearly 70 °C with stirring. Then 0.1 M thiourea (H_2_N-CS-NH_2_) solution was added dropwise up to complete the reaction as well as precipitation. The precipitate was washed away frequently with DI water and then kept in a dryer to remove the moisture part. Finally, the PbS nanoparticles were obtained via calcination at two different temperatures, 300 °C, and 360 °C for 6 h. The detail method is schematically depicted in Figure 1.

X-ray diffraction (XRD) spectroscopy has been employed to investigate the structural properties of the PbS nanoparticles. The XRD patterns were taken in the 2θ ranging from 20° to 60° using the ‘BRUKER aXS-D8 Advance Cu-Ka’ diffractometer. The FESEM model ‘LEO 1450 Vp’ has been used for investigating the morphology as well as grain size and growth of the PbS nanoparticles. For TEM imaging, a drop of PbS solution was deposited on a form varcoated grid for transmission electron microscopy using a Talos L120C. The grid was loaded via a sample holder for picture acquisition after air drying. UV-vis spectrometer ‘Perkin Elmer Instruments Lambda35′ was used to measure optical characteristics such as optical transmittance, absorbance, and bandgap. The carrier concentration, carrier mobility, and resistivity were all measured using the ECOPIA 3000 Hall measurement system.

In addition, PbS thin films on FTO coated and bare glass substrate has been fabricated using the Spin-coating technique, and films’ structural, optical and electrical properties have been investigated. Also, the influence of PbS thin-film characteristics on the device performance of planar perovskite solar cells was investigated using SCAPS-1D, a well-known solar cell device modeling program (PSCs). The potential application of PbS in PSC has been realized using various factors including PbS HTL layer parameters discovered in this work. The cell output performance was assessed using the light current-voltage (I–V) and capacitance-voltage (C–V) characteristics. The optoelectrical properties of PbS thin films have been found to have a substantial impact on PSCs, which could aid in the construction of high efficiency and very stable PSCs.

## 3. Results and Discussion

**Figure 2:**
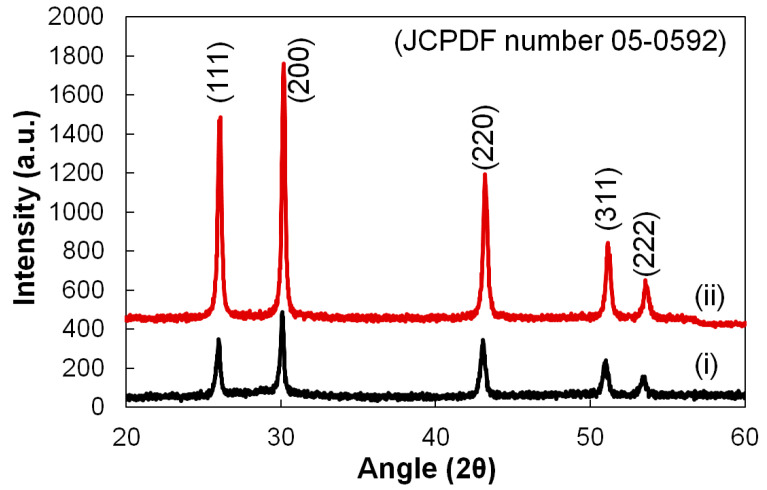
XRD pattern of PbS nanoparticles synthesized via plant-extract supported method, (i) for 300 °C and (ii) is for 360 °C of calcination temperature.

The XRD patterns of the prepared PbS nanoparticles for 300 °C and 360 °C of calcination temperature are shown in Figure 2. It could be seen that both synthesized nanoparticles have a similar X-ray diffraction pattern including the same indices and the pattern corresponds to cubic PbS (PDF number 05-0592) [39,40]. However, the peak intensity along the (200) plane is higher for 360 °C of calcination temperature indicating that a better quality PbS nano-particle could be achieved via this process. The average crystal sizes (D) were estimated based on the peak width of the (200) planes by using Scherrer’s formula [41]:

(1) $$D=\frac{0.94\lambda }{\beta Cos\theta }$$

From Table 1, it could be seen that the average crystallite size of the nanoparticles is larger at 360 °C of calcination temperature which corresponds with the increases in XRD peaks constricting. Besides, the micro-strain, *ε*, and dislocation density, *δ* has also been estimated using the formula mentioned in the literature [42]:

*ε* = β/4tanθ (2)

*δ* = 1/D^2^ (3)

The estimated values of *ε* and *δ* are shown in Table 1. Particularly, *ε* and *δ* can be influenced by the substitution and/or relocation of atoms in the films as well as by the promiscuous grain distribution. Hereby, we found that the *ε* and *δ* for the plane (200) are higher for PbS film prepared at a lower temperature or 300 °C of calcination temperature. It may be occurred due to a decrease in grain size and grain distribution during the crystallization process during the calcination. Besides, as shown in the next section, the films are S atom rich, which may impact the increased dislocation density. Also, the increase of *ε* and *δ* may have occurred due to the replacement of Pb^2+^ and S^2−^ atoms in their reference lattice during the calcination process.

**Figure 3:**
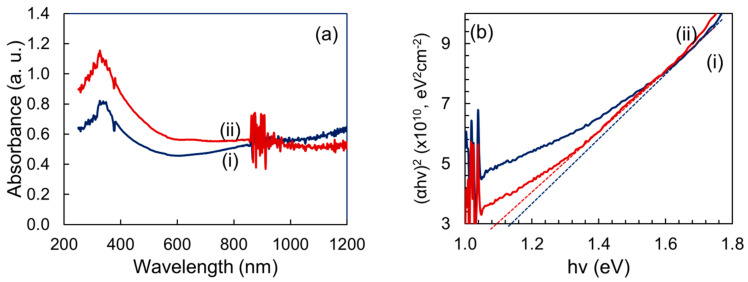
(**a**) Absorbance and (**b**) Tauc plots of PbS nanoparticles synthesized via plant-extract supported method, (i) for 300 °C, and (ii) for 360 °C of calcination temperature.

Figure 3a shows the absorption spectrum of PbS nanoparticles which was recorded at room temperature within the range of 200 nm–1200 nm. There is a peak observed at around 320 nm for both films which can be assigned to the exciton transitions [43]. The variation of the absorbance in the films may be related to the particle size. Usually, a reduction in particle size causes an increase in the bandgap and consequently, a blue shift occurs. The bandgap energies were estimated by extrapolation of Tauc plots [41] are shown in Figure 3b. It has been seen that the bandgap of PbS particle synthesis using 300 °C is 1.12 eV which is 0.03 eV higher than PbS synthesis at 360 °C signifies that the quantum confinement is much stronger in PbS nanoparticles prepared at a lower temperature [44]. Particularly, the decrease in particle size, as well as the increase in bandgap energy of the as-prepared nanoparticles, signifies the size quantization effects [45]. Size quantization of the charges in a small volume crystallite is well known for forming the blue shift.

**Figure 4:**
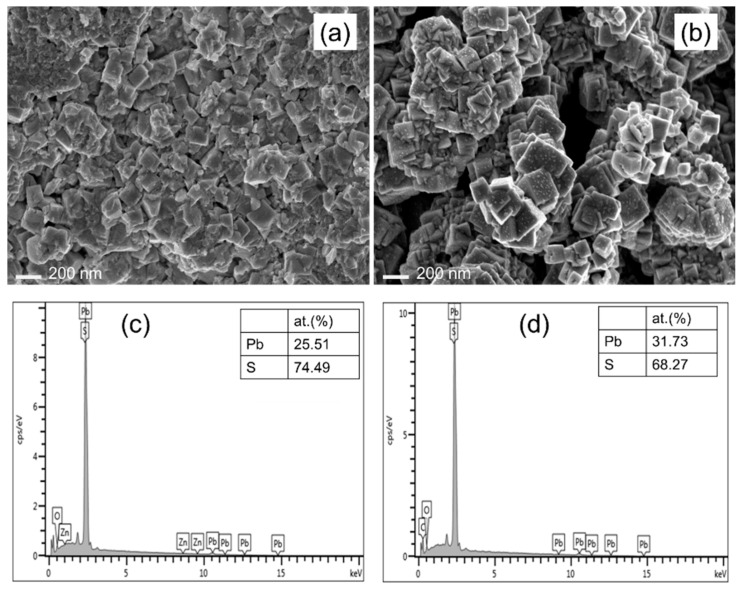
(**a**,**b**) SEM images and (**c**,**d**) EDX spectra of PbS nano-particles synthesized via plant-extract supported method, (**a**,**c**) is for 300 °C and (**b**,**d**) for 360 °C of calcination temperature.

SEM analysis is used for examining the morphology and size of PbS nanoparticles as shown in Figure 4. It is shown that the PbS has no definite structure; some of them are found in the form of stereo structure and some of them in the form of a polygon. The image shows that the grain size is ranging 100–200 nm where bigger grains are observed for 360 °C of calcination temperature. Also, it could be observed that the particles are aggregated in both cases. Temperature, in particular, is conducive to nanoparticle nucleation and growth, and particle size changes as a result. Due to the high surface energy and aggregation, particle size may also rise with temperature. The surface energy of the particles rose as the calcination temperature increased, resulting in increasing aggregation. As a result, the bigger particles were produced. Similar tendencies have also been reported in the synthesis of NiO [46] and TiO_2_ [47]. Figure 4c,d show the atomic composition of PbS films. It could be seen that both films are non-stoichiometric and S rich which may have an impact on the film’s crystallography and optical properties as seen in XRD and UV-Vis results. A small percentage of O atoms have been detected which may be diffused during the calcination process.

**Figure 5:**
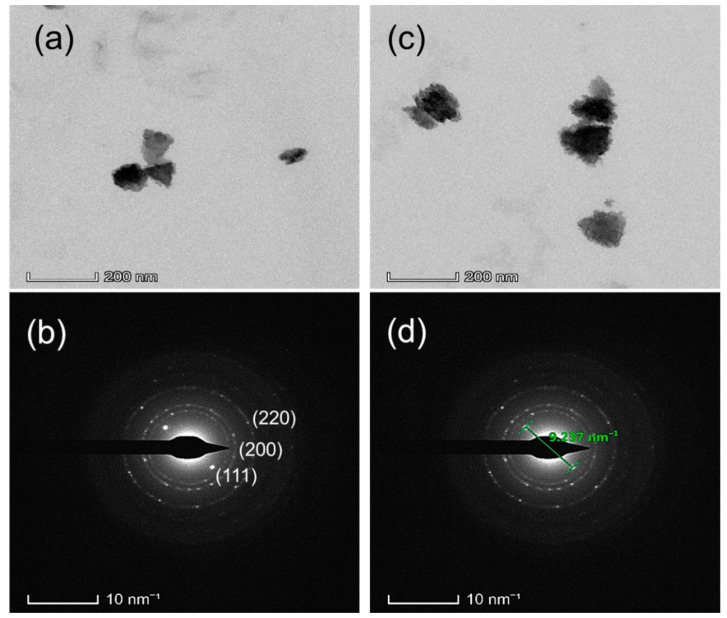
TEM and SAED images of PbS nanoparticles synthesized via plant-extract supported method, (**a**,**b**) are for 300 °C, and (**c**,**d**) is for 360 °C of calcination temperature.

Figure 5 shows TEM images that verify the creation of PbS nanoparticles in typical size ranges of 35 to 200 nm, confirming the findings in SEM, where a bigger particle size was found for 360 °C of calcination temperature as it was seen in the SEM image also. Particularly, the TEM images can give information about the particle sizes, particle distribution, lattice imperfection, and the homogeneity of the nanoparticles of materials on the atomic scale. Figure 5 confirms that the nanoparticles are not round-shaped and also they are not distributed homogeneously. The prepared PbS nanoparticles have polydispersity in particle size, as shown by the rings of the selected area electron diffraction (SAED) pattern (Figure 5b,d). Furthermore, the SAED pattern reveals concentric rings with bright spots, indicating that the PbS NPs are nanocrystalline and have good crystalline properties. The SAED patterns were indexed to correspond with the cubic PbS rock-salt structure’s (111), (200), and (220) planes, and the diffraction rings corresponded well with the corresponding XRD patterns of the PbS nanoparticles [48,49]. All of the above characterization results confirm the formation of lead sulfide (PbS) nanoparticles.

## 4. Thin Film Fabrication and Device Modeling

**Figure 6:**
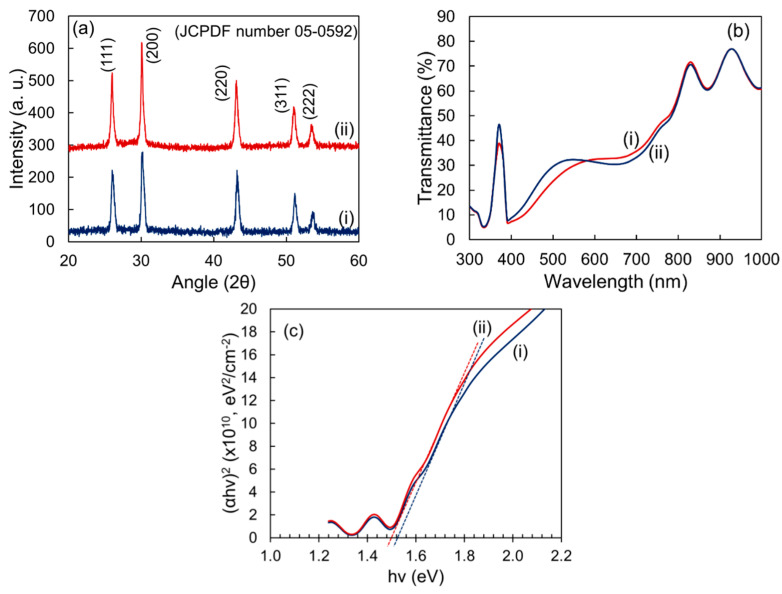
(**a**) XRD patterns, (**b**) transmittance spectra, and (**c**) Tauc plot of the thin film fabricated from the two different solution concentrations of PbS nanoparticles, (i) 5 mg mL^−1^ and (ii) 10 mg mL^−1^.

The green synthesized PbS nanoparticles of 360 °C calcination temperature were dissolved in Toluene with concentrations of the solution 5 mg mL^−1^ and 10 mg mL^−1^. The solutions were stirred for 24 h and a light-black colloidal suspension was obtained. PbS films were deposited on clean FTO coated and bare glass substrates by spin-coating. The substrates were clean using sonication that has been demonstrated elsewhere [42]. About 50 μL of ink was dropped with a micropipette at the center of the substrate and the chuck was rotated at 1000 rpm for 10 s and then, 3000 rpm for 20 s. The precursor film was then heated at 100 °C in the air for around 20 min to evaporate excess solution and brown PbS films are found. The structural and optical properties, such as XRD spectra, transmittance, absorbance, and the bandgap of the films for two different solution concentrations are shown in Figure 6.

It could be seen in Figure 6a that all peaks of the fabricated films are similar to the peaks observed previously for nanoparticles, however, the intensity of all of the peaks is reduced drastically. On the other hand, the XRD peak intensity, as well as transmittance and absorbance of the films, indicate the effect of the solution concentration. The variation observed in the structural and optical properties may lead by the nano-particle orientation, film surface, and thickness. The bandgap of the films is found to be 1.52 eV for solution concentration of 5 mg mL^−1^ and 1.48 for solution concentration of 10 mg mL^−1^, respectively. It could be easily predicted that the film fabrication using the solution of higher concentration may be thicker than the film fabricated using lower concentration which leads to slightly better crystalline properties as seen in Table 2 and, consequently, the bandgap is reduced (as observed in Figure 6c). Similar properties have also been reported by Vankhade and Chaudhuri [50] who studied details of thickness-dependent properties of spin-coated nano-crystalline PbS thin films. Using the ECOPIA 3000 Hall-Effect measurement system, the electrical characteristics of nano-crystalline PbS thin films prepared on a glass substrate were examined. The measured carrier concentration, mobility, and resistivity are shown in Table 2. It could be seen that the film prepared using 10 mg mL^−1^ of solution concentration showed higher mobility than the film prepared using 5 mg mL^−1^ solution. This higher mobility acquired by the films may be due to higher crystalline properties, such as low dislocation densities.

**Figure 7:**
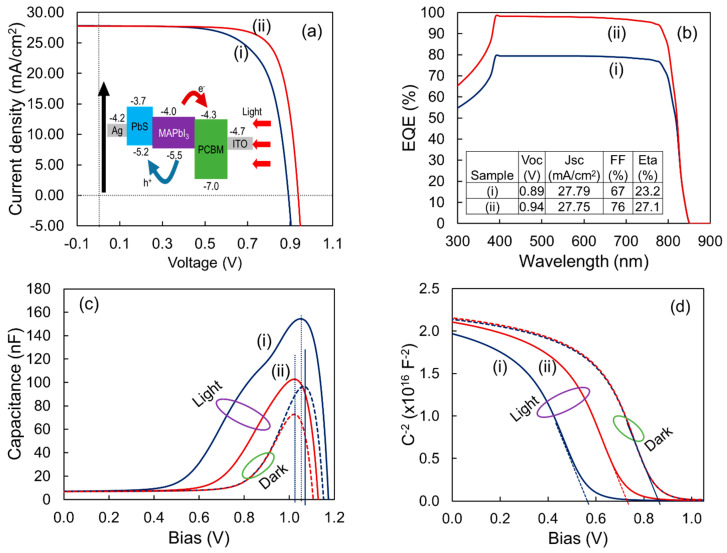
(**a**) The light current density-voltage (J–V) curves found for PSCs with different PbS thin films, (**b**) shows the quantum efficiency (QE) for the PSCs, (**c**,**d**) shows the light and dark capacitance-voltage (C–V) curves for the modeled PSCs (inset: (**a**) shows the schematic band diagram and (**b**) shows the performance parameters of modeled PSCs and (i) and (ii) is indicate the PSCs with two different PbS films that have been deposited using 5 mg mL^−1^ and 10 mg mL^−1^ of solution concentration).

The performance of the PSC device was studied using the one-dimensional programme SCAPS-1D (version 3.3.01) and PbS as a hole transport layer (HTL). The optoelectrical properties of PbS were employed in this investigation, with the exception that the thickness was set to 80 nm. Readers are referred to the literature for more information on SCAPS-1D, including other layer parameters [51,52]. The energy band diagram of the modeled PSC structure is shown in the inset of Figure 7a. Figure 7a,b, and the inset Table show the light current-voltage (J–V) characteristics and quantum efficiency (QE) of the simulated PSCs using different electrical and optical properties of the PbS thin films that have been found in this study. It could be seen that Voc and FF varied significantly with the change of optical and electrical properties of the PbS thin films. It is well known that FF is dependent on the carrier transport and extraction that occurred in PSC. Additionally, the transport and carrier extraction in the solar cells depends on the mobility and the morphology of the films as well as the bulk and interfacial carrier recombination rates. Furthermore, the Voc of PSC is based on the splitting of the quasi-Fermi energy levels of the hole and electron in the entire system, according to the traditional p-i-n semiconductor model. As a result, the energy distributions of perovskite thin films, as well as the charge transport materials, have a major impact on PSC performance [53,54].

Besides, the measurements of capacitance-voltage (C–V) in both dark and light situations were investigated to determine the effect of PbS materials on the built-in potential (V_bi_) of PSCs. According to the standard Mott-Schottky model, the V_bi_ is estimated by the intercept based on the CV curves as shown in Figure 7c,d [55,56]. The bulk band offset in solar cells and the energy differential between the interfaces of distinct layers, in particular, govern the V_bi_. The V_bi_ in PSCs determines the energy differential at the cathode/ETL and anode/HTL interfaces, as well as bulk polarization from grain boundary defects [56]. Furthermore, in PSCs such as the metal-insulator-metal (MIM) model, the energy differential at the interface of cathode/ETL and anode/HTL controls the V_bi_ in the case of the passivated grain boundary. As observed in this work, the features of the carrier transport layer have a significant influence on the V_bi_ of PSCs. As shown in Figure 7d, the V_bi_ of the PSCs with different PbS HTLs fabricated in this study are determined to be 0.55 V to 0.76 V under light (one-sun) conditions, interestingly 0.87 V for both in dark conditions. The effect of the PbS materials’ optical and electrical properties on the device V_bi_ in both dark and light circumstances is seen here.

Additionally, due to the accumulation of photo-generated carriers at different interfaces, the cathode/ETL and anode/HTL interfaces may contribute to photoexcitation. Consequently, the V_bi_ may be separated into dynamic and static components based on the energy difference and buildup of photo-generated carriers at the cathode/ETL and anode/HTL interfaces. The dynamic component, particularly the accumulation of photo-generated carriers at their interface, has a significant impact on the V_bi_ [57,58]. The Vpeak shift exhibited in Figure 7c for films with solution concentrations of 5 mg mL^−1^ and 10 mg mL^−1^ can reflect interfacial photo-generated carrier buildup in solar cells [41]. The density of the accumulated photo-generated carriers at the interface could be determined by the height of the Vpeak. This indicates that for the film of 10 mg mL^−1^ solution concentration, there was less interface-charge accumulation, resulting in a greater V_bi_ via a dynamic parameter in the PSC. It can be inferred that PSC efficiency is strongly reliant on the features of PbS thin films, implying that PbS HTL requires much more research to get the most effective and stable PSCs. PbS thin film could surely be a potential HTL than other materials currently used in PSCs, based on the simulated highest efficiency of 27.1%.

## 5. Conclusions

We successfully synthesized PbS using Aloe Vera extract in a simple, green, cost-effective as well as time-effective method for producing good quality PbS nanoparticles. The prepared nanoparticles were investigated in detail employing XRD, UV-Vis, FESEM, TEM, and SAED studies. The experimental results show that the calcination temperature could affect the crystal structure of the final products. The XRD and SAED patterns are indicated the synthesized PbS nanoparticles are cubic rock-salt structures with the (111), (200), and (220) planes. It has also been found that the crystallite sizes, micro-strain, and dislocation densities of the PbS increase as the calcination temperature increases. The FESEM and TEM images confirm that the nanoparticles are not round-shaped and even not distributed homogeneously. The SAED pattern’s rings indicate that the produced PbS nanoparticles have good crystallinity and polydispersity in particle size. The structural properties were almost retained while fabricating PbS thin films using the Spin coating technique. It has been found that the structural and optoelectrical properties are dependent on the PbS nanoparticle solution concentration. The film properties have been implemented for simulating planar perovskite solar cells. Device simulation shows that the best device properties are found for the 10 mgmL^−1^ solutions of PbS nanoparticles. The C–V analysis shows that the film prepared using 10 mg mL^−1^ solution concentration assure a better interface with the perovskite and neighbor metal contact. The highest PCE of 27.1% has been obtained via numerical simulation including Voc = 0.94 V, Jsc = 27.75 mA cm^−2^ and FF = 76%. The findings show that PbS nanoparticles could be a potential HTL in the fabrication of high-efficiency and stable perovskite solar cells.

## Figures and Tables

**Table 1:** Estimated structural properties of the PbS nanoparticles with respect to the calcination temperature.

	Process Temperature	Peak Position, (200) (2θ°)	Crystallite Size, (nm)	Microstrain, *ε* (×10^−3^)	Dislocation Density, *δ* (×10^14^ cm^−2^)
(i)	300 °C	30.08	24.21	5.52	17.07
(ii)	360 °C	30.20	31.66	4.20	9.97

**Table 2:** Estimated structural and electrical properties of the PbS thin films with respect to the solution concentration.

	Peak Position, (111) (2θ^o^)	Crystallite Size, (nm)	Microstrain, *ε* (×10^−3^)	Dislocation Density, *δ* (×10^14^ cm^−2^)	Carrier Con. (cm^−3^)	Mobility (cm^2^ V^−1^ s^−1^)	Resistivity (×10^2^) (Ω-cm)
(i)	30.22	17.89	7.43	31.22	4.1 × 10^14^	43	9.17
(ii)	30.06	30.48	4.39	10.76	10.6 × 10^14^	54	7.11

## Data Availability

Data will be available from the corresponding author upon request.
